# Effectiveness of art therapy on pain and anxiety in children undergoing invasive medical procedures

**DOI:** 10.6026/973206300213843

**Published:** 2025-10-31

**Authors:** Bhasara Kalpana Ramji, Mahalakshmi B, Siva Subramanian N

**Affiliations:** 1 Department of Pediatric Nursing, Nootan College of Nursing, Sankalchand Patel University, Visnagar, Gujarat - 384315, India; 2 Department of Psychiatric Nursing, Nootan College of Nursing, Sankalchand Patel University, Visnagar, Gujarat - 384315, India

**Keywords:** Art therapy, pediatric pain, anxiety; invasive medical procedures, non-pharmacological intervention

## Abstract

The effectiveness of art therapy in reducing pain and anxiety in children aged 3-12 undergoing invasive medical procedures is of interest. Hence, a one-group pretest-posttest design involved 41 participants receiving a 20-25 minute art therapy session. Results showed significant reductions in pain (mean scores from 8.90 to 5.22) and anxiety (mean scores from 9.24 to 5.27) post-intervention. Severe pain levels decreased from 97.6% to 12.2%, while severe anxiety dropped from 90.2% to 26.8%. Thus, we show art therapy as a beneficial non-pharmacological intervention in pediatric care.

## Background:

Hospitalization and medical procedures can be profoundly distressing experiences for children. The unfamiliar environment, separation from parents and fear of pain often evoke significant anxiety and discomfort, particularly during invasive procedures [1]. In pediatric healthcare, addressing procedural pain and anxiety is vital not only for the child's immediate well-being but also for long-term psychological outcomes. Unmanaged procedural distress can lead to medical trauma, treatment noncompliance and increased sensitivity to pain in future encounters [2]. Non-pharmacological interventions like art therapy have gained attention as holistic, child-centered strategies to alleviate procedural distress [3]. Art therapy engages children in creative processes such as drawing, painting, or coloring, providing distraction and an expressive outlet for emotions. This modality is particularly suitable for pediatric care due to its accessibility, low cost and developmental appropriateness [4]. Numerous studies have documented the efficacy of art-based interventions in managing pain and anxiety in pediatric populations. For example, a randomized controlled trial using the TICK-B coloring intervention demonstrated significant reductions in venipuncture-related pain and anxiety in children aged 6-12. Similarly, scoping reviews suggest consistent emotional benefits, including reductions in stress, fear and pain intensity [5]. Art therapy has also shown promise across diverse medical settings. For example, in pediatric oncology, integrative approaches such as art, music and play therapies were associated with meaningful symptom control during chemotherapy and surgery [6]. Another review found that over 80% of cancer patients experiencing pain turned to complementary interventions like art therapy to manage symptoms [7]. Despite growing support, studies on art therapy in procedural contexts remain limited in pediatric populations. Many focus on chronic conditions or emotional health outcomes rather than acute procedural anxiety and pain [8]. The present study aims to contribute to this field by evaluating the impact of a short, structured art therapy session on pain and anxiety in children undergoing invasive medical procedures. Therefore, it is of interest to describe the effectiveness of art therapy on pain and anxiety in children undergoing invasive medical procedures.

## Methodology:

## Research design and setting:

The study employed a one-group pretest-posttest design to assess the effectiveness of art therapy on reducing pain and anxiety among children undergoing invasive medical procedures. The study was conducted in the pediatric units of Sneh Children Hospital, Modhera Chokadi, Mehsana and Krishna Children Hospital, Radhanpur Road, Mehsana.

## Population and sample:

The population included children aged 3-12 years undergoing invasive medical procedures. A total of 41 children were enrolled using convenience sampling.

## Intervention:

The intervention consisted of a 20-25 minute art therapy session where children were encouraged to draw or color freely using provided materials. The researcher facilitated the process but did not influence creativity. Sessions were conducted just before or during procedures, functioning both as distraction and emotional expression.

## Data collection instruments:

[1] Demographic profile of children and parents.

[2] Pain measurement: Wong-Baker Faces Pain Rating Scale (0-10), categorized into mild, moderate and severe.

[3] Anxiety measurement: Observational Scale of Behavioral Distress (Jay & Elliott, 1984) scored 0-10 and categorized as mild, moderate, or severe.

## Data analysis:

Data were analyzed using SPSS. Descriptive statistics summarized demographic variables. Paired t-tests compared pre- and post-test scores, with significance set at p < 0.05. Results showed a significant reduction in mean pain (8.90 → 5.22) and anxiety (9.24 → 5.27), confirming the effectiveness of art therapy. Ethical clearance was obtained from the institutional review board. Informed consent was taken from parents and assent from children when possible. Confidentiality and voluntary participation were assured throughout.

## Results:

Table 1 (see PDF) shows the demographic profile of children in the art therapy group, where the majority were aged 3-5 years (39.0%), males (56.1%) and mostly cared for by mothers (92.7%). Table 2 (see PDF) demonstrates the associations between demographic variables and post-test outcomes, with age (χ^2^=12.880, p=0.042), family income (χ^2^=7.881, p=0.048) and hospitalization history (χ^2^=8.921, p=0.012) significantly related to pain reduction, while gender (χ^2^=7.421, p=0.025) and surgical history (χ^2^=9.214, p=0.010) were significantly associated with anxiety reduction. Figure 1 (see PDF) depicts the pre-test and post-test distribution of pain and anxiety categories, showing that severe pain (97.6%) and anxiety (90.2%) before therapy reduced to 12.2% and 26.8% after therapy, with most children shifting into moderate or mild categories, confirming the positive effect of art therapy.

## Discussion:

This study demonstrated that art therapy significantly reduced both pain and anxiety in children undergoing invasive medical procedures. Severe pain levels dropped from 97.6% to 12.2% and severe anxiety from 90.2% to 26.8%, highlighting the effectiveness of art therapy as a non-pharmacologic intervention. Our results align with prior evidence. In an RCT, art-based distraction during venipuncture reduced pain and anxiety significantly [9]. Adolescents treated with art therapy in emergency care also showed 23-28% reductions in pain and anxiety [10]. In post-operative care, art therapy lowered anxiety and behavioral distress compared to routine care [11]. These outcomes align closely with existing scientific literature. Mersal *et al.* (2025) demonstrated that art-based play interventions during venipuncture substantially reduced both physiological and emotional stress markers in hospitalized children [12]. Similarly, Olaizola *et al.* (2024) conducted a scoping review that confirmed art therapy's positive role in pediatric pain modulation and emotional resilience [5]. Goren *et al.* (2023) found that creative modalities including music, play and art meaningfully alleviated pain and anxiety in hospitalized pediatric patients [13]. Chiang *et al.* (2023) showed that art and play interventions helped mitigate trauma-related distress in children with chronic or acute medical histories [14]. In a landmark study, Favara-Scacco *et al.* (2001) observed reduced psychological trauma and pain perception in children undergoing painful procedures such as lumbar punctures, following structured art therapy sessions [15]. Lastly, Tsze and Bifano (2024) reported 20-30% reductions in procedural pain and anxiety scores following art therapy exposure in pediatric emergency departments [10]. Together, these studies strongly validate our findings, reinforcing that art therapy is a low-risk, high-impact intervention capable of significantly reducing procedural pain and anxiety in pediatric care. Its simplicity, affordability and adaptability across settings make it especially valuable in both high- and low-resource medical environments. In pediatric oncology, combined art and music therapy improved mood, coping and social outcomes [16], while art therapy improved emotional resilience and communication in long-term cancer care. Creative tasks reduce attention to pain via distraction mechanisms. Expression through art also helps children externalize fears they cannot verbally articulate [17]. In cancer settings, art therapy was shown to improve treatment compliance and reduce emesis, pain and anxiety [18]. We found stronger responses among younger children, those with no prior hospitalizations and from lower-income backgrounds-groups often more susceptible to procedural distress. This supports similar findings on integrative therapies benefiting underserved populations. Girls and first-time surgical patients also showed greater anxiety reduction, matching evidence that such populations are more responsive to distraction-based therapy [19]. Emerging digital tools like tablet-based art apps also show promise as alternatives or adjuncts to traditional therapy. Music and virtual reality therapies have shown parallel effects in reducing cortisol and stabilizing vital signs during procedures [20]. Goren *et al.* (2023) found high-certainty evidence supporting play therapy and moderate support for music in pain and anxiety reduction [13]. Art therapy offers a low-cost, accessible and effective solution to procedural pain and anxiety in children. It is especially beneficial for younger, vulnerable, or first-time patients and should be integrated into pediatric procedural protocols. Future research should compare art therapy to other modalities, explore long-term outcomes and examine mechanisms of change through mixed methods.
